# Correlation Analysis of *Staphylococcus aureus* Drug Resistance and Virulence Factors with Blood Cell Counts and Coagulation Indexes

**DOI:** 10.1155/2023/8768152

**Published:** 2023-02-15

**Authors:** Jing Wei, Kaihui Ma, Yuan Zhang, Xincheng Yang, Qiao Tang, Zhenlin Nie

**Affiliations:** ^1^Department of Laboratory Medicine, Nanjing First Hospital, Nanjing Medical University, Nanjing 210006, Jiangsu, China; ^2^Department of Clinical Laboratory, Yancheng Maternity and Child Health Hospital, 31 Century Avenue East Road, Yancheng 224000, Jiangsu, China

## Abstract

**Objective:**

The influence of different *Staphylococcus aureus* variants on blood cells and coagulation system was evaluated by investigating the carrying status of drug resistance genes and virulence genes of methicillin-resistant*Staphylococcus aureus* (MRSA) and methicillin-sensitive*Staphylococcus aureus* (MSSA).

**Methods:**

A total of 105 blood culture-derived*Staphylococcus aureus* strains were collected. The carrying status of drug resistance genes mecA and three virulence genes *tst*, *pvl,* and *sasX* was analyzed by polymerase chain reaction (PCR). The changes in routine blood routine counts and coagulation indexes of patients infected with different strains were analyzed.

**Results:**

The results showed that the positive rate of mecA was consistent with that of MRSA. Virulence genes *tst* and *sasX* were detected only in MRSA. Compared with MSSA, patients infected with MRSA or MSSA patients infected with virulence factor, leukocyte count and neutrophil count in peripheral blood were significantly increased, and the platelet count decreased to a higher degree. Part thromboplastin time increased, D-dimer increased, but fibrinogen content decreased more. The changes of erythrocyte and hemoglobin had no significant correlation with whether *Staphylococcus aureus* carried virulence genes.

**Conclusion:**

The detection rate of MRSA in patients with positive *Staphylococcus aureus* in blood culture had exceeded 20%. The detected MRSA bacteria carried three virulence genes, *tst*, *pvl,* and *sasX*, which were more likely than MSSA. MRSA, which carries two virulence genes, is more likely to cause clotting disorders.

## 1. Introduction

The drug resistance rate of *Staphylococcus aureus* (*S. aureus*) increased year by year [1]. Due to the multiple drug resistance and strong pathogenicity, community-associatedmethicillin-resistant*S. aureus* (CA-MRSA) has been a serious health threat for the past 25 years [2]. Among them, atopic dermatitis and soft tissue infection are the most common. When they are derived from the skin and mucous membrane, they have the ability to produce sepsis and highly fatal pulmonary infection, such as necrotic pneumonia, purpura, and toxic shock syndrome [3]. *mecA* is almost universal in MRSA isolates and is usually resistant to many types of nonlactam antibiotics [4]. About the pathogenicity of *S. aureus*, there are three common virulence factors: toxic shock toxin-1 encoded by the *tst*, Panton–Valentine leukocidin encoded by the *pvl*, and targeting surface protein *sasX* encoded by the *sasX* [5].

The routine diagnostic test for *S. aureus* infection is a blood cell test. Because the *S. aureus* in the infected site will stimulate the immune cells in the patient to secrete chemokines, making the white blood cells in the bone marrow of the patient release into the blood, the patients often have an abnormal hemogram, that is, the white blood cells and neutrophils are significantly increased. The number of white blood cells is generally (10–30) × 10^9^/L, with a shift to the left in the nuclear index, toxic granules appearing in the cells, and the number of eosinophils decreasing [6].

The common systemic infections caused by *S. aureus* are sepsis. Sepsis occurs when a *S. aureus* infection invades the bloodstream, multiplies, and produces toxins that cause symptoms of systemic poisoning. After the occurrence of sepsis, a large amount of *Staphylococcus aureus* in the blood for reproduction forms bacterial emboli, which will spread to all tissues and organs of the human body along with blood circulation, forming suppurative metastasis. Sepsis will invade the microcirculation of the victim, causing septic shock and multiple organ failure, and life-threatening in severe cases [7].

It has been reported that when patients develop sepsis caused by *S. aureus*, they often have poor coagulation function. With the deep research on *Staphylococcus aureus*, the effect of *S. aureus* on blood coagulation function has received immense attention. Studies have found that the severity of sepsis in patients is related to the degree of coagulation dysfunction. If the patient has local tissue trauma of the skin and *S. aureus* infection, often because of the bleeding at the trauma cannot be solved in time, thus aggravating the degree of damage to the body, it is usually treated with dialysis [8].

Sepsis with *S. aureus* infection is associated with coagulopathy, which is essentially a change in blood quality. The main mechanism is that the complex of teichoic acid and peptidoglycan in the cell wall of *S. aureus* activates the complement system in the same way that the endotoxin of Gram-negative bacteria activates the complement system and causes inflammation. The activation of the complement system can promote the release of vasoactive amine, induce abnormal activation of the coagulation system, weaken the effect of anticoagulation pathway, and the body is in a state of hypercoagulation, and a large number of coagulation factors are abnormally consumed, leading to the occurrence of disseminated intravascular coagulation (DIC) [9]. Subsequently, multiple bleeding, microthromboembolism, shock, and impaired function of various organs may be followed. At this point, the patients will have abnormalities in four indicators: prothrombin time (PT), activated partial thromboplastin time (APTT), thrombin time (TT), fibrinogen level (FIB), platelet count (PLT), and D-dimer (D-D).

At present, studies on *S. aureus* and coagulation system are limited to this, and there are few reports on the relationship between drug resistance and virulence-related molecules and coagulation function. By searching for the relationship between the two, we can help find out the mechanism of *S. aureus* affecting the coagulation function in other aspects and help to improve the body damage caused by the abnormal coagulation function in the patients with sepsis caused by *S. aureus*.

## 2. Materials and Methods

### 2.1. Materials

A total of 105 *S. aureus* strains were collected, all of which were isolated from blood samples of outpatients and inpatients in the Second Affiliated Hospital of Suzhou University. The isolates were isolated from January 25, 2013, to July 19, 2018, and the repeated isolates were eliminated. Data were checked for normality using Kolmogorov–Smirnov and Shapiro–Wilk tests. The bacterial identification and drug sensitivity test were conducted by the American BD company Phoenix100 automatic bacteria identification/drug sensitivity system. The quality control strain was *Staphylococcus aureus* (ATCC25923). The oligos were synthesized by ShengGong Company.

### 2.2. Bacterial Culture

*Staphylococcus aureus* strains stored at −80°C were inoculated in a 5 mL LB liquid medium, shaken at 37°C (180°r/min) for overnight culture (12–14°h), then mixed and inoculated on blood plate, and incubated at 37°C for 16–18°h. Single colonies were selected by an inoculation ring and transferred to blood plate again. After incubation at 37°C for 16–18°h, purified *S. aureus* colonies were obtained. A single colony was selected, and *Staphylococcus aureus* was confirmed again by a bacterial mass spectrometer (BD Bruker MALDI-Typer™). MALDI Biotyper™ uses MALDI-TOF (matrix-assisted laser desorption ionization time-of-flight mass spectrometry) to obtain characteristic molecular fingerprints of microorganisms and use them as a basis for microbial identification.

### 2.3. DNA Preparation and PCR

We add 100 *μ*L of sterilized deionized water into a 0.5 mL Eppendorf tube, select a single colony into the tube, add 2 mg/mL Lysostaphin 3 *μ*L, vortex mix, put in a 37°C water bath for 30 min, and then set the temperature to 100°C boil for 30 min.

Centrifugation was performed at 12000 rpm for 5 min at low temperature; then, the supernatant was absorbed and placed in a new Eppendorf tube, and then sterilized deionized water was added for 10 times dilution, which was the extracted DNA template of *S. aureus*.

PCR was performed using Dream Taq Green PCR Master Mix (2x). The PCR cycling protocol consisted of predenaturation at 95°C for 5 min, 35 cycles of denaturation at 95°C for 30°s, annealing at 55°C for 30°s, extension for 90°s, and final extension at 72°C for 8 min. The amplification products were analyzed by agarose gel electrophoresis. The primer pairs used for PCR are as follows: *mecA* (forward: 5′-AAAATCGATGGTAAAGGTTGGC-3′ and reverse: 5′-AGTTCTGCAGTACCGGATTTGC-3′);*sasX* (forward: 5′-AGAATTAGAAGTACGTCTAAATGC-3′ and reverse: 5′-GCTGATTATGTAAATGACTCAAATG-3′);*pvl* (forward: 5′-GTGCCAGACAATGAATTACCC-3′ and reverse: 5′-TTCATGAGTTTTCCAGTTCACTTC-3′);*tst* (forward: 5′-TTCACTATTTGTAAAAGTGTCAGACCCACT-3′ and reverse: 5′-TACTAATGAATTTTTTTATCGTAAGCCCTT-3′); 16S rRNA (forward: 5′-AGAGTTTGATCCTGGCTCAG-3′ and reverse: 5′-GGTTACCTTGTTACGACTT-3′).

### 2.4. Statistical Analysis of Data

The experimental data are analyzed statistically using the Student's *t* test for two-treatment comparisons. *p* < 0.05 is considered as significant.

## 3. Results

### 3.1. Drug Resistance of *Staphylococcus aureus*

**Figure 1:**
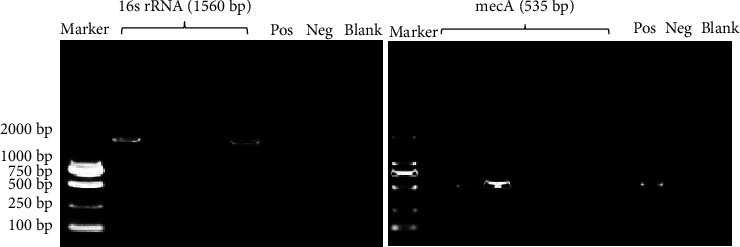
Partial PCR amplification bands 16s rRNA and *mecA*.

Gene *mecA* is one of the most common genes responsible for drug resistance in *S. aureus* [10]. A total of 22 strains containing *mecA* were detected by PCR in the 105 strains of *S. aureus* collected. The detection rate of *mecA* was 20.95%. Some results of PCR amplification of 16S (rRNA reference gene) and *mecA* in *S. aureus* are shown partly (Figure 1).

The 105 isolates of *S. aureus* were tested for drug sensitivity. Among them, 25 cases were diagnosed as MRSA (23.8%). The results of drug sensitivity showed that 21 of the 22 *mecA* positive strains were MRSA, which were resistant to penicillin, cephalosporins, and *β*-lactamase inhibitor compounds. Among them, one *mecA* positive strain showed drug sensitivity as MSSA, and among the 83 *mecA* negative strains, 4 strains showed drug sensitivity as MRSA, while the rest showed as MSSA (Table 1). Most MRSA carry *mecA,* which is consistent with the reported results [11, 12]. As can be seen from the percentage value in Table 1, the positive rate of *mecA* was generally consistent with the positive rate of MRSA (*p* < 0.001). Only a few strains showed inconsistency between *mecA* gene carrying and MRSA determination. Four MRSA strains did not carry *mecA*, and one MSSA strain carried *mecA*.

Statistical analysis of drug sensitivity results of 25 strains of MRSA to 19 different antimicrobial agents (Table 2) showed that MRSA was resistant to most antimicrobial agents, and resistant to all penicillin, cephalosporins, and other *β*-lactam antibiotics, such as cefoxitin, penicillin, and oxacillin, with 100% resistance rate. Among them, there were 10 antimicrobial drugs with a drug resistance rate above 40%. Except the above 3 antimicrobial drugs with 100% drug resistance, the other antimicrobial drugs were clindamycin (84%), erythromycin (84%), tobramycin (64%), tetracycline (48%), gentamicin (48%), ciprofloxacin (48%), and amikacin (40%) in the order. According to the antimicrobial classification, these MRSAs were resistant to macrolides, tetracyclines, quinolones, and aminoglycosides. MRSA was sensitive to the following antibiotics, including rifampicin, furantoin, mupirocin, teicoplanin, vancomycin, and linezolid, with a sensitivity rate of 100%, and no resistant strains were found. There were 9 antibiotics with a sensitivity rate of more than 70%. Except for antibacterial drugs with a sensitivity rate of 100%, the sensitivity rates were quinupristin/dalfopristin (96%), bactrim (92%), and trimethoprim (76%) in the order. According to the classification of antimicrobial agents, these MRSAs were sensitive to rifamycin, sulfonamides, nitrofurans, oxazolidinones, and glycopeptides.

**Figure 2:**
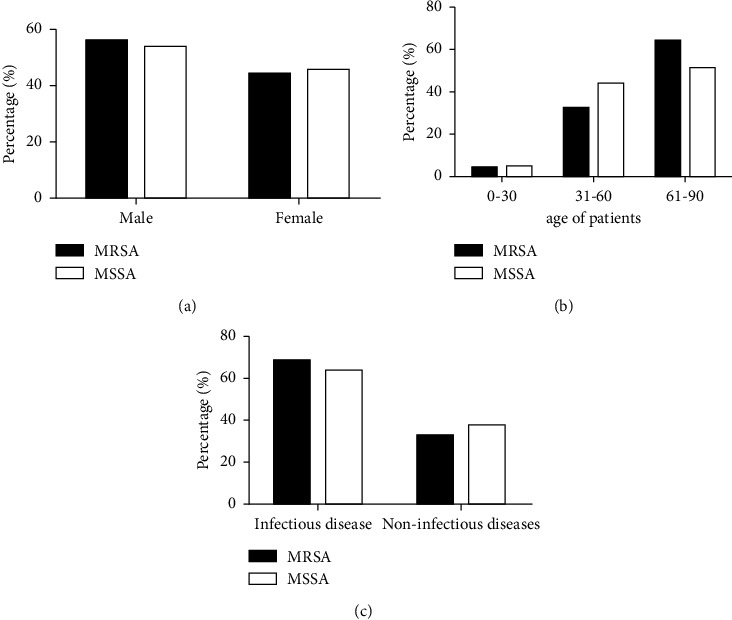
Comparison of basic information of patients infected with MRSA or MSSA. (a) Basic patient information was disaggregated by gender. (b) By age. (c) By whether or not they are infectious diseases.

In order to understand the basic information of patients infected with MRSA or MSSA, this paper compared and analyzed the distribution of patients infected with the two types of SA from three aspects, including gender (Figure 2(a)), age (Figure 2(b)), and clinical diagnosis (Figure 2(c)). The three statistical graphs all showed that there was little difference in gender, age, and clinical diagnosis distribution between MRSA- and MSSA-infected patients, which undoubtedly greatly increased the reliability of subsequent studies.

### 3.2. The Leukocyte Count and Neutrophil Count in Peripheral Blood of Patients Infected with MRSA Were Significantly Increased

Bacterial infection often leads to increased leucocyte count reactivity [13]. In this paper, we retrospectively analyzed the blood routine indexes of 25 patients infected with MRSA and 80 patients infected with MSSA and compared the white blood cell count, neutrophil count, lymphocyte count, red blood cell count, and hemoglobin content, respectively. The changes of eosinophils and basophils were also analyzed, but there was no statistical difference in their counts between patients infected with MRSA and MSSA. Therefore, the data and analysis graphs will be ignored below.

**Figure 3:**
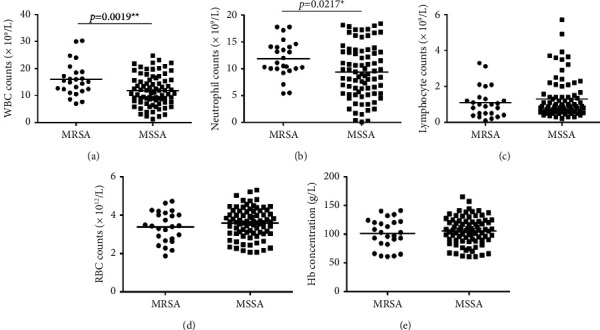
Comparison of blood count of patients infected with MRSA or MSSA. (a) WBC counts. (b) Neutrophil counts. (c) Lymphocyte counts. (d) RBC count. (e) Hb concentration.

As shown in Figure 3(a), compared with MSSA infected patients, the number of white blood cells in the peripheral blood of MRSA infected patients is significantly increased (Figure 3(a), *p* < 0.01). By counting neutrophils and lymphocytes separately, it can be seen that the number of neutrophils in patients infected with MRSA increases significantly compared with those infected with MSSA (Figure 3(b), *p* < 0.05), while the number of lymphocytes decreases more, but there is no statistical difference (Figure 3(c)). The statistics show that the resistance of *S. aureus* to methicillin had little correlation with RBC number (Figure 3(d)) and hemoglobin content (Figure 3(e)).

### 3.3. Patients Infected with MRSA Have Abnormal Peripheral Blood Coagulation Indexes

**Figure 4:**
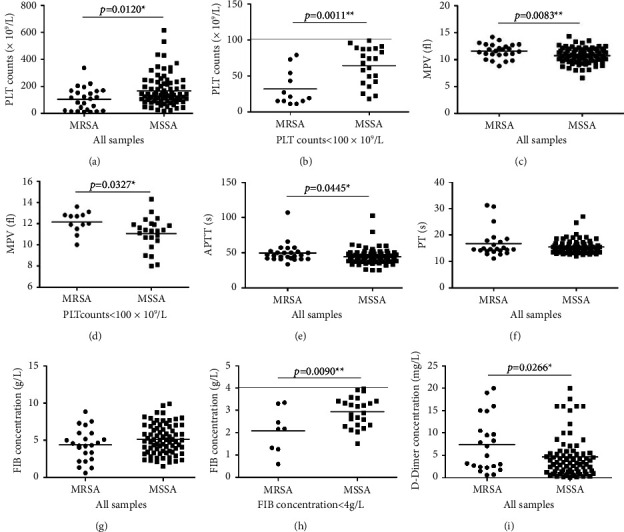
Comparison of peripheral blood coagulation indexes of patients infected with MRSA or MSSA. (a) PLT counts (all samples). (b) PLT counts (thrombocytopenia patient). (c) MPV (all samples). (d) MPV (thrombocytopenia patient). (e) APTT. (f) PT. (g) FIB concentration (all samples). (h) FIB concentration (fibrinogenopenia patient). (i) D-dimer concentration.

Patients with bacteremia, especially those with severe sepsis, often experience a sharp drop in platelet counts. Studies have shown that platelet count in ICU sepsis patients is correlated with patient survival rate [14]. In addition, severe bacterial infection can cause disseminated intravascular coagulation (DIC), resulting in the death of the patient [15]. Therefore, it is of great clinical significance to analyze the correlation between MRSA or MSSA and coagulation indicators. The results showed that compared with MSSA-infected patients, the platelet count in the peripheral blood of MRSA-infected patients was significantly decreased (Figure 4(a), *p* < 0.05). When the PLT count was less than 100 × 10^9^/L, patients infected with MRSA showed more significant thrombocytopenia (Figure 4(b), *p* < 0.01). When the number of platelets in the peripheral blood decreases, the new platelets are accelerated to enter the peripheral blood for supplementation. The volume of newborn platelets is relatively large, so the detection of mean platelet volume (MPV) can reflect the formation of newborn platelets. According to statistics, the MPV of patients infected with MRSA was larger than that of patients infected with MSSA. The conclusion is consistent when the PLT count of the whole sample is counted (Figure 4(c), *p* < 0.01), or only when the PLT count of the sample is less than 100 × 10^9^/L (Figure 4(d), *p* < 0.05).

Moreover, the APTT of MRSA patients was higher than that of MSSA patients (Figure 4(e), *p* < 0.05). We can see the same trend about PT, but the difference is not statistically significant (Figure 4(f)).

Bacterial infection often causes hyperfibrinolysis, leading to a large consumption of fibrinogen and production of D-dimer, leading to clotting disorders [16]. The scatter diagram shows lower fibrinogen (FIB) levels in blood samples infected with MRSA than those infected with MSSA. When all full samples were counted, the FIB concentration in MRSA-infected blood samples was correspondingly low, but the difference is not statistically significant (Figure 4(g)). In the crowd of fibrinogenopenia patients, the FIB content in the MRSA-infected blood samples was significantly lower than that of the MSSA-infected blood samples (Figure 4(h), *p* < 0.01). Moreover, the content of D-dimer in blood samples infected with MRSA was significantly higher than that infected with MSSA (Figure 4(i), *p* < 0.05).

### 3.4. Virulence of *Staphylococcus aureus*

**Figure 5:**
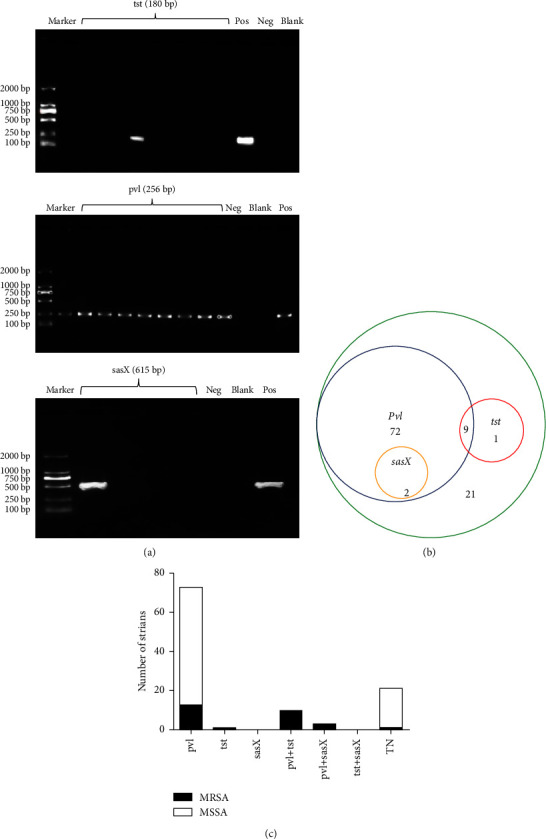
Distribution of virulence factors carried by MRSA and MSSA. (a) The conventional RT-PCR assay of expression of virulence genes *tst*, *pvl,* or *sasX.* (b) Distribution diagram of virulence genes *tst*, *pvl,* and *sasX.* (c) Histogram of *tst*, *pvl,* and *sasX* distribution of MRSA or MSSA.

Virulence genes *tst*, *pvl,* and *sasX* are the most common virulence genes in *S. aureus* [17]. In this study, the abovementioned three virulence genes of *S. aureus* isolated from peripheral blood of 105 patients with bacterial infection are detected by PCR amplification (Figure 5(a)). Among 105 strains of *S. aureus*, 83 strains carried the virulence gene *pvl*, and the carrying rate was 79%. A total of 10 strains carrying the virulence gene *tst* are detected, and the carrying rate was 9.5%. Only 2 strains carried the virulence gene *sasX*, with a carrying rate of 2%. Carrying rate from high to low: *pvl*, *tst*, and *sasX*.

Among the 83 *Staphylococcus aureus* strains carrying *pvl*, 9 strains carried *tst,* and 2 carried *sasX* simultaneously. Strains carrying the virulence gene *sasX* all carried *pvl*. Among the 105 strains of *S. aureus*, none carried three virulence genes at the same time, that is, there is no triple-positive strains. Nine strains which carried *pvl* and *tst* were called double-positive strains. The other two double-positive strains carried *pvl* and *sasX*. There are 11 double-positive strains, 73 single-positive strains, and 21 triple-negative strains (Figure 5(b)).

MRSA and MSSA are compared for carrying different virulence genes (Table 3). All the 10 strains carrying the virulence gene *tst* were MRSA. Two strains carrying *sasX* were also MRSA. Among the 83 strains carrying virulence gene *pvl*, 23 were MRSA and 60 were MSSA. The positive rate of *pvl* in MRSA was 92%, and only two MRSA strains were *pvl* negative. The carrying rate of *tst* and *sasX* in MRSA was significantly higher than that in MSSA (*p* < 0.05). However, there was no significant difference between MRSA and MSSA in the carrying rate of *pvl* (*P*=0.068). All the 11 double-positive strains carrying two virulence factors are MRSA, while among the 21 triple-negative strains without any virulence factors, only one is MRSA and the other 20 strains are MSSA (Figure 5(c)). In conclusion, MRSA is more likely to carry virulence genes than MSSA, especially *tst* and *sasX*.

### 3.5. The Leukocyte Count and Neutrophil Count in Peripheral Blood of Patients Infected with *S. aureus* Increased with More Virulence Factors

*Staphylococcus aureus* that does not carry the above three virulence genes is defined as triple-negative*Staphylococcus aureus*, a total of 21 cases. A total of 73 *S. aureus* cases with only one virulence gene were defined as single-positive*S. aureus*, among which 72 cases carried only *pvl* virulence gene and just one case carried only *tst* virulence gene. *S. aureus* strains carrying virulence genes *pvl* and *tst* or *pvl* and *sasX* were called double-positive*S. aureus*, and 11 cases in total. The differences of blood cell counts and clotting indexes in peripheral blood of patients infected with single-positive (SP), double-positive (DP), or triple-negative (TN) *S. aureus* were compared. In order to better explore the correlation between *Staphylococcus aureus* virulence factors and the above indicators, we regrouped the *S. aureus* variants according to certain rules. The single-positive *S. aureus* mainly carried the virulence gene *pvl*, and the double-positive*S. aureus* mainly carried the virulence gene *pvl* and also carried one of *tst* and *sasX*, among which the *S. aureus* mainly carried both *pvl* and *tst*. When single-positive*S. aureus* was compared with triple-negative*S. aureus*, it could also be regarded as an analysis of the virulence gene *pvl*. When *S. aureus* DP was compared with *S. aureus* SP or TN, the main research is the effects of *sasX* and *tst* genes, especially *tst*.

**Figure 6:**
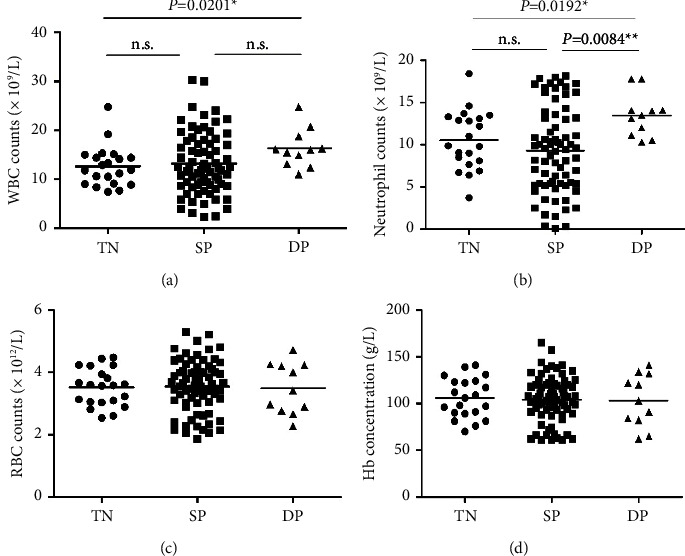
Comparison of peripheral blood cell counts in patients infected with *S. aureus* with different virulence factors.

There was no significant difference in the number of peripheral white blood cells in the *S. aureus* SP patients compared with the double-positive and triple-negative*S. aureus* patients. It can also be said that the virulence gene *pvl* has no correlation with the number of white blood cells in patients. However, Figure 6 shows that the number of peripheral blood white blood cells in patients with double-positive*S. aureus* infection is significantly higher than that in patients with triple-negative infection (Figure 6(a), *p* < 0.05) and single-positive*S. aureus* infection.

The results showed that the number of neutrophils in the peripheral blood of the patient is mainly affected by the virulence factor carried by *S. aureus* (Figure 6(b)), among which the number of neutrophils was significantly increased by the virulence factor *tst* carried by *S. aureus*.

With MRSA and MSSA about blood correlation analysis, we found that different virulence factor of *S. aureus* infection bacteria in patients with peripheral blood red blood cell count and hemoglobin content, see from the statistical data whether carry one or two virulence factors did not affect the patient number of red blood cells in peripheral blood (Figure 6(c)) and hemoglobin content (Figure 6(d)).

### 3.6. *S. aureus*-Infected Patients with More Virulence Factors Have Abnormal Peripheral Blood Coagulation Indexes

**Figure 7:**
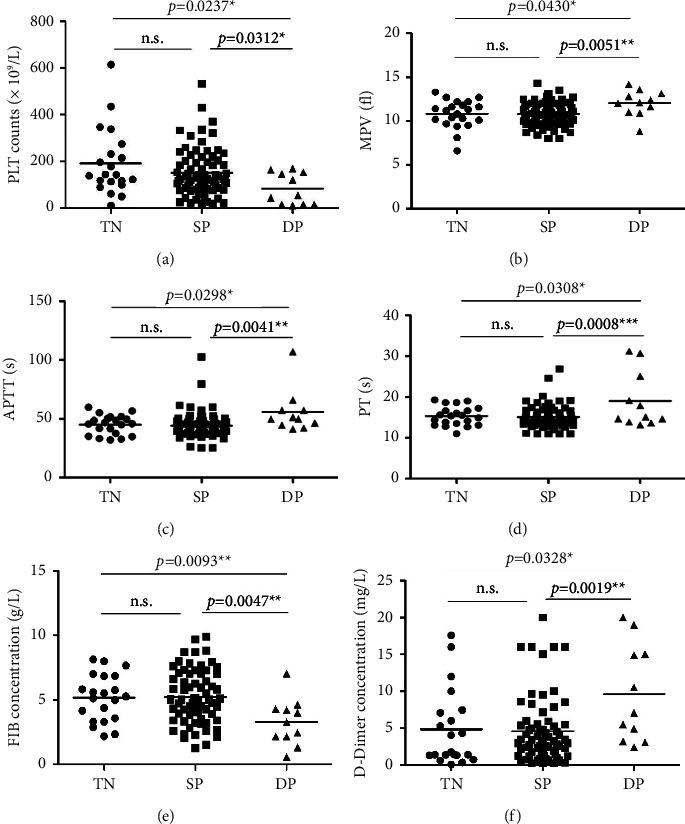
Comparison of peripheral blood coagulation indexes in patients infected with *S. aureus* with different virulence factors.

The platelet content in peripheral blood of double-positive*S. aureus*-infected patients carrying two virulence genes was lower than that of *S. aureus*SP-infected patients and *S. aureus*TN-infected patients. Statistical *P* values showed that the virulence genes *tst* and *sasX* have a greater effect on platelet count, while the virulence gene *pvl* has the opposite effect (Figure 7(a)). In addition, the mean volume of platelets in the peripheral blood of patients with double-positive*S. aureus* was significantly increased (Figure 7(b)).

APTT and PT indicators in peripheral blood of infected patients with one virulence gene, two virulence genes, and no virulence gene were counted, respectively, and no difference was found between the three-negative and single-positive*S. aureus* groups. However, when double-positive*S. aureus* was compared with single-positive*S. aureus* and triple-negative*S. aureus* infection patients, the results showed that the PT and APTT in the infected *S. aureus* strains with both virulence genes are significantly prolonged (Figures 7(c) and 7(d)).

The contents of fibrinogen and D-dimer in the peripheral blood of patients infected with triple-negative, single-positive, and double-positive*S. aureus* were detected. Except *pvl*, other virulence genes were highly correlated with the fibrinogen content and D-dimer content in the peripheral blood of patients. In other words, the fibrinogen content in the peripheral blood of *S. aureus*-infected with both virulence genes is significantly decreased and the D-dimer content is significantly increased compared with that of *S. aureus*-infected with no virulence gene or only one virulence gene (Figures 7(e) and 7(f)).

## 4. Discussion

At present, MRSA has become one of the main pathogens of nosocomial infection in inpatients, and bloodstream infection caused by MRSA is an important reason for the high mortality of infectious diseases [18]. With the increasing detection rate of MRSA, drug resistance is gradually enhanced, which is a great challenge for clinical treatment. Drug resistance and pathogenicity are two important biological properties of pathogenic bacteria [19]. We focused on the correlation between the drug resistance and virulence of *S. aureus* strains and blood cells and coagulation function in the blood samples from which the strains originated, so as to understand the situation of peripheral blood and coagulation function of patients infected with *S. aureus* and to lay a foundation for the treatment of *S. aureus* infection.

It has been reported that MSSA and MRSA show great differences in the types of virulence factors secreted. MSSA usually produces inflammatory lysotoxin A, G, D, and interleukin (PVL) [20]. MRSA produces more TSST-1, but both MSSA and MRSA can produce these toxins, just in different proportions. In 1982, Schlievert et al. found that vaginal mucosal MRSA produced less toxin A and more TSST-1 than skin MRSA [21]. It has been speculated that high levels of cytolysin are needed to produce inflammation, destroy the integrity of the skin barrier, and predispose to infection with *S. aureus*. In contrast, the production of the same amount of cytolysin on the upper surface of the mucosa leads to a strong host response that quickly dies from a severe infection or the microorganism is immediately eliminated by the host immune system. These indicate that the production of virulence factors varies greatly among different hosts. We made statistics of the virulence gene *tst*, *pvl,* and *sasX* in MRSA and MSSA, respectively, and the results showed that *tst* and *sasX* were more likely to be carried in MRSA than MSSA, while whether *pvl* was carried had no correlation with whether it was sensitive to methicillin. From the composition of triple-negative and double-positive strains, MRSA had a higher probability of carrying virulence factor than MSSA.

Toxic shock syndrome (TSS) is a multiorgan disease characterized by rash, fever, and hypotension with rapid onset. The disease was mainly caused by TSS toxin-1 (TSST-1) produced by *S. aureus*. TSST-1 belongs to the same superantigen family as enterotoxin secreted by *S. aureus* and exotoxin secreted by streptococcus [22]. Bacterial superantigens selectively interact with V*β* on T cell receptor (TCR), thereby inducing a large number of peripheral T cells to proliferate and secrete proinflammatory cytokines [23]. MRSA carrying *tst* will have a great impact on the immune system of patients.

Plenty of literature has reported that platelets not only participate in the process of hemostasis and thrombosis, but also play an important role in the innate immune and inflammatory responses of the body. The correlation between inflammation and blood clotting has gradually been discovered [24]. When inflammation occurs, a large number of inflammatory cells infiltrate, macrophages and T lymphocytes activate and promote the increase of inflammatory factors. Inflammatory factors can promote the production of platelet activation factors and platelet activation. Platelet activating factor (PAF) can also act as a strong inflammatory mediator to exert chemotaxis on white blood cells [25]. It can be seen that inflammation and platelet activation have mutually promoting effects. Platelets serve as an immune defense in the event of infection, inflammation, or sepsis [26]. Platelets are activated and consumed too much, so infection is often accompanied by a decreased platelet count and bleeding. In conclusion, inflammatory factors can activate platelets, resulting in thrombocytopenia, decreased coagulation function, and hyperactivity of fibrinolytic system, which is consistent with the changes of coagulation-related indicators in the blood samples of MRSA and *S. aureus* carrying virulence genes in the paper.

At present, *S. aureus* has been widely studied, but the effect of *S. aureus* on peripheral blood cells and coagulation function of patients is still unclear. Due to the insufficient number and range of collected strains, the statistical samples were limited, the distribution of the three virulence factors was uneven, and the detection rate of virulence genes *tst* and *sasX* was low, which may lead to the phenomenon difference. The mechanism of the above conclusions will be further confirmed.

## 5. Conclusion

In conclusion, the detection rate of MRSA in patients with blood-borne infection of *S. aureus* was more than 20%, and more than 80% of MRSA patients carried drug resistance gene *mecA*, which was resistant to all penicillins, cephalosporins, and other *β*-lactam antibiotics. MRSA is more likely to carry both virulence genes than MSSA. Compared with MSSA carrying virulence gene *pvl*, patients infected with MRSA carrying multiple drug-resistant genes were more likely to have changes in the number of white blood cells, neutrophils, platelets, and abnormal blood coagulation indexes. The results may provide an experimental basis for the clinical diagnosis and treatment of *S. aureus* infection.

## Figures and Tables

**Table 1:** The *mecA* carried in MRSA and MSSA.

*mecA*	MRSA (*n* = 25)	MSSA (*n* = 80)	*P* value
	% (*n*)	% (*n*)	
Positive	84% (21)	1.25% (1)	*P* < 0.001
Negative	16% (4)	98.75% (79)	

**Table 2:** Drug sensitivity of 25 strains of MRSA to antimicrobial agents.

Antibacterial agents	Sensitive strains	Sensitive rate	Intermediary strains	Intermediary rate	Resistant strains	Resistant rate
Amikacin	11	0.44	4	0.16	10	0.40
Cefoxitin	0	0.00	0	0.00	25	1.00
Clindamycin	4	0.16	0	0.00	21	0.84
Rifampicin	25	1.00	0	0.00	0	0.00
Tetracycline	13	0.52	0	0.00	12	0.48
Bactrim	23	0.92	0	0.00	2	0.08
Furantoin	25	1.00	0	0.00	0	0.00
Oxacillin	0	0.00	0	0.00	25	1.00
Quinupristin/dalfopristin	24	0.96	0	0.00	1	0.04
Mupirocin	25	1.00	0	0.00	0	0.00
Ciprofloxacin	12	0.48	1	0.04	12	0.48
Erythromycin	4	0.16	0	0.00	21	0.84
Teicoplanin	25	1.00	0	0.00	0	0.00
Tobramycin	9	0.36	0	0.00	16	0.64
Vancomycin	25	1.00	0	0.00	0	0.00
Linezolid	25	1.00	0	0.00	0	0.00
Gentamicin	13	0.52	0	0.00	12	0.48
Penicillin	0	0.00	0	0.00	25	1.00
Trimethoprim	19	0.76	0	0.00	6	0.24

**Table 3:** Comparison of virulence gene carrying between MRSA and MSSA.

Virulence genes	*MRSA (n* *=* *25)*		*MSSA (n* *=* *80)*		*P* value
	Positive number (*n*)	Positive rate (%)	Positive number (*n*)	Positive rate (%)	
*Tst*	10	40	0	0	≤0.001
*Pvl*	23	92	60	75	0.068
*sasX*	2	8	0	0	0.011

## Data Availability

The data used to support the findings of this study are available from the corresponding author upon reasonable request.
